# The Impact of COVID-19 on Blood Glucose: A Systematic Review and Meta-Analysis

**DOI:** 10.3389/fendo.2020.574541

**Published:** 2020-10-05

**Authors:** Juan Chen, Chunhua Wu, Xiaohang Wang, Jiangyi Yu, Zilin Sun

**Affiliations:** ^1^Department of Endocrinology, Jiangsu Province Hospital of Chinese Medicine, Affiliated Hospital of Nanjing University of Chinese Medicine, Nanjing, China; ^2^Department of General Practice, Zhongda Hospital, Medical School, Southeast University, Nanjing, China; ^3^Department of Endocrinology, Zhongda Hospital, Institute of Diabetes, Medical School, Southeast University, Nanjing, China

**Keywords:** COVID-19, glucose, glycated hemoglobin A1c, systematic review, meta-analysis

## Abstract

**Background:** Diabetes mellitus is considered a common comorbidity of COVID-19, which has a wide spectrum of clinical manifestations ranging from asymptomatic infection to severe respiratory symptoms and even death. However, the impact of COVID-19 on blood glucose has not been fully understood. This meta-analysis aimed to summarize available data on the association between glycemic parameters and severity of COVID-19.

**Methods:** PubMed, EMBASE, and Cochrane Library were searched from December 1, 2019 to May 15, 2020. Observational studies investigating blood glucose or glycated hemoglobin A1c (HbA1c) according to the severity of COVID-19 were considered for inclusion. Two independent researchers extracted data from eligible studies using a standardized data extraction sheet and then proceeded to cross check the results. Data were pooled using a fixed- or random-effects model to calculate the weighted mean differences (WMDs) and 95% confidence intervals (CIs).

**Results:** Three studies reported blood glucose and HbA1c according to the severity of COVID-19 and were included in this meta-analysis. The combined results showed that severe COVID-19 was associated with higher blood glucose (WMD 2.21, 95% CI: 1.30–3.13, *P* < 0.001). In addition, HbA1c was slightly higher in patients with severe COVID-19 than those with mild COVID-19, yet this difference did not reach significance (WMD 0.29, 95% CI: −0.59 to 1.16, *P* = 0.52).

**Conclusions:** This meta-analysis provides evidence that severe COVID-19 is associated with increased blood glucose. This highlights the need to effectively monitor blood glucose to improve prognosis in patients infected with COVID-19.

## Introduction

Coronavirus disease 2019 (COVID-19), caused by severe acute respiratory syndrome coronavirus 2 (SARS-CoV-2), has a wide spectrum of clinical manifestations ranging from asymptomatic infection to severe respiratory symptoms, and even death (1). According to recent researches, the overall mortality of COVID-19 ranged from 1.4 to 15% (2–4). Furthermore, an epidemiological study from Italy reported that the mortality of patients requiring intensive care unit admission was 26% (5). Of note, once the disease progresses to acute respiratory distress syndrome, the mortality would be ~40% (6). Because of efficient person-to-person transmission, it has become an emerging worldwide threat for human health.

Currently, several studies have been designed to focus on details of the clinical and virological course of SARS-CoV-2 infection. Subsequently, studies suggest that individuals with older age and comorbidities, such as diabetes and hypertension, are more likely to have COVID-19, as well as a higher risk of mortality (2, 3, 7). As we all know, diabetes mellitus is considered a public health concern worldwide due to its increasing prevalence and involvement in the development of several diseases, including stroke, kidney failure, and heart disease (8). Since diabetes has been reported to be associated with poor prognosis of COVID-19, glycemic management for patients with both diabetes and COVID-19 has gained much more attention (9, 10). There are evidences that better glycemic control is closely associated with improvement in clinical outcomes in COVID-19 patients (4, 11). Nevertheless, at the same time, it is confused that whether COVID-19 contributes to hyperglycemia. A previous study suggested that pancreas could be the target of coronavirus attack since SARS-CoV was detected in pancreas (12). Furthermore, another research found SARS-CoV damaged the endocrine part of pancreas, indicating that SARS-CoV may cause acute insulin dependent diabetes mellitus (13). In addition, it is worth noting that infection leads to profound alterations in whole-body metabolism, including glucose, fat, and protein (14). Although many studies suggest that diabetes is an important risk factor for COVID-19. It remains unclear regarding the effect of severity of COVID-19 infection on glycemic parameters, including blood glucose and glycated haemoglobinA1c (HbA1c).

At present, the rapid worldwide spread of COVID-19 requires continual improvement of knowledge about glycemic management during COVID-19 infection. Therefore, we conducted this meta-analysis by incorporating the latest evidence with focus on association between glycemic parameters and severity of COVID-19, which may be instructive for clinical practice.

## Methods

### Search Strategy

An extensive search strategy was designed on PubMed, Embase and the Cochrane Library to retrieve all articles published from December 1, 2019 to May 15, 2020, following the MOOSE group guidelines of observational meta-analyses (15). No language restrictions were applied. The details of the search strategy are available in the Supplementary Table 1. The primary outcome measure was blood glucose or glycated haemoglobinA1c (HbA1c). Two authors (JC and CHW) independently reviewed the titles and abstracts to identify potentially relevant articles. After being identified as relevant articles, the full texts were individually analyzed by both authors, independently, to determine whether the article was qualified for eligibility criteria. During the study selection process, disagreements were resolved through consultation with a third investigator (JYY). In addition, the reference lists of included studies, relevant review articles and meta-analyses were screened for other suitable studies to maximize the search for articles on the same topic.

### Study Selection and Quality Assessment

Studies were considered eligible if they fulfilled the following criteria: (1) participants were diagnosed with COVID-19; (2) separate data for patients with mild and severe COVID-19 infection (those who required mechanical ventilation, intensive care unit admission or those who died) were provided; (3) information on any of the prespecified primary outcomes were provided. If suitable data were not available or unclear in the published papers, the corresponding authors were contacted to request this information. Studies were excluded if they were conference abstracts, editorials, commentary, case reports, reviews, nonhuman studies, or did not expressly report the values of glycemic parameters in COVID-19 patients according to the severity. The quality of studies was assessed using the Newcastle-Ottawa Scale (16). We rated cohort studies a maximum of four stars for selection, two stars for comparability, and three stars for outcome assessment. Disagreements were resolved by discussion between two authors or consulting a third investigator (JYY) if necessary.

### Data Extraction

Two independent reviewers (JC and CHW) extracted data from eligible studies using a standardized data extraction sheet and then proceeded to cross check the results. Disagreements between two reviewers regarding extracted data were resolved by discussion or consulting a third investigator (JYY) if necessary. The following information was extracted: first author name, publication year, country, sample size, study design, age, gender, primary outcomes including blood glucose and HbA1c.

### Data Synthesis and Statistical Analysis

For studies reporting interquartile range, the standard deviations were obtained using the methods described in Cochrane Handbook for Systematic Reviews. The weighted mean differences (WMDs) with 95% confidence intervals (CIs) were calculated. The heterogeneity was evaluated using the Q test and *I*^2^ statistic. *I*^2^ > 50% or *P* < 0.1 was considered to have a significant heterogeneity. A fixed-effects model was used when the result showed no significant heterogeneity, otherwise a random-effects model was applied. All the analyses were conducted using STATA software (Version 12.0, StataCorp LP, College Station, Texas). A 2-sided *P* < 0.05 was set for statistical significance.

## Results

### Study Selection and Characteristics

**Figure 1:**
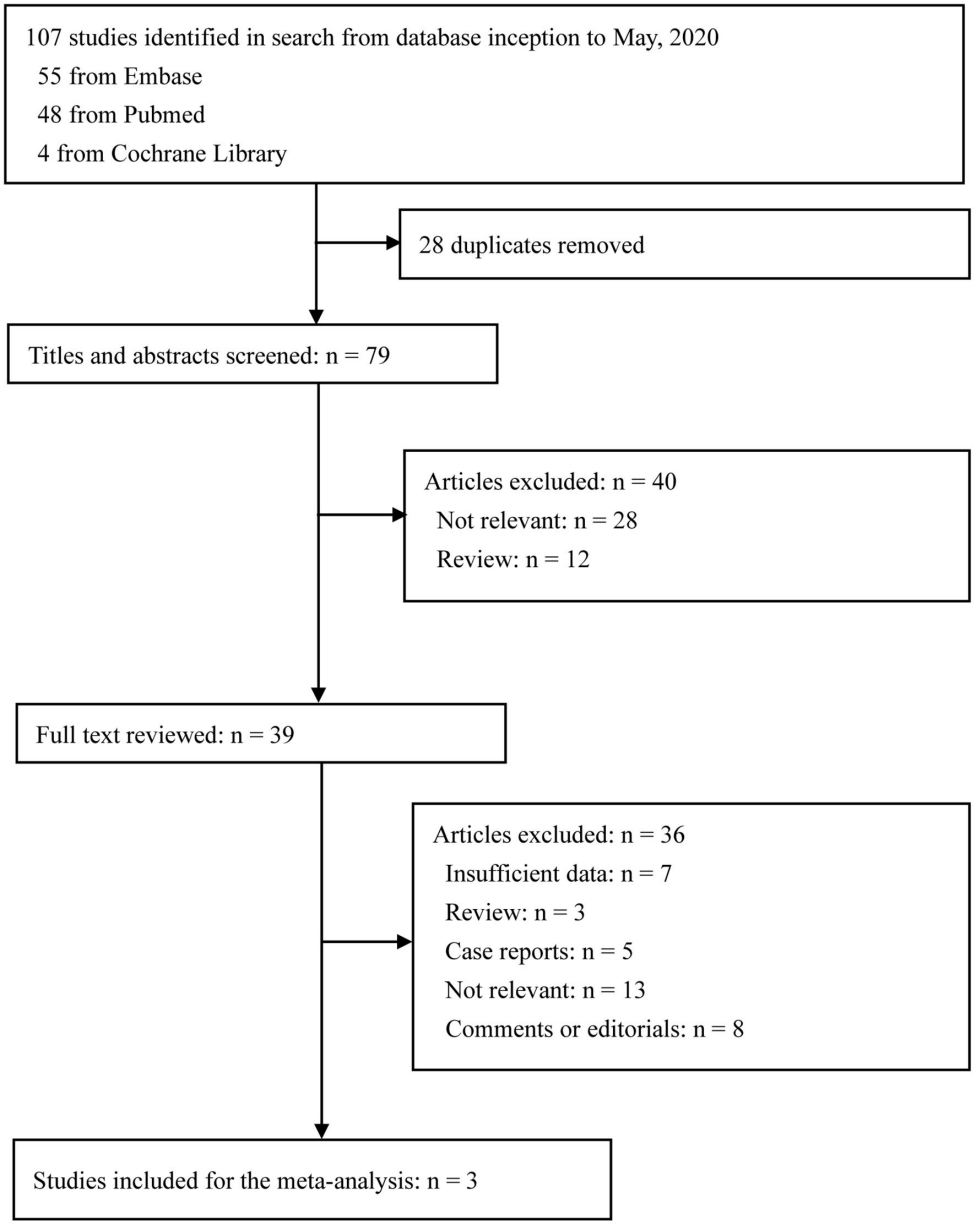
Flow diagram of study selection.

The literature search result and study selection process are presented in Figure 1. Overall, 107 citations of interest were found in the initial electronic searches of PubMed, Embase, and the Cochrane Library. After excluding 28 duplicates, 79 potentially eligible articles were selected. Of these, 39 full text papers were potentially relevant and assessed for eligibility. Finally, three papers were included in the meta-analysis that evaluated blood glucose and/or HbA1c levels according to the severity of COVID-19 (17–19).

Of the three included studies, two articles reported data on both blood glucose and HbA1c, one article only reported data on blood glucose, and all studies used retrospective design. Study sample sizes ranged from 28 to 151, with a total of 222 COVID-19 patients, including 131 patients in mild group and 91 in severe group. Compared with women, men were more likely to have COVID-19 infection. Moreover, patients with severe COVID-19 were older than those with mild COVID-19. The detailed characteristics of the studies included in the meta-analysis are presented in Table 1.

**Table 1:** Characteristics of studies included in this meta-analysis.

**Study**	**Period**	**Study type**	**Country**	**No**.	**Age (year)**	**Male (%)**	**BG (mmol/L)**	**HbA1c (%)**	**Diabetes (%)**
Ren et al. (17)	January 12, 2020 to February 13, 2020	Retrospective cohort study	China	151	Mild case: 53.9 ± 16.2 Severe case: 67.6 ± 11.6	78 (52%)	Mild case: 6.2 ± 1.7 Severe case: 8.4 ± 4.3	Mild case: 6.0 ± 1.5 Severe case: 6.7 ± 2.1	Mild case: 16 (18.0%) Severe case: 23 (37%)
Wang et al. (18)	January 29, 2020 to February 10, 2020	Retrospective cohort study	China	28	Mild case: 65.8 ± 9.4 Severe case: 71.4 ± 7.9	21 (75%)	Mild case: 9.8 ± 3.4 Severe case: 13.7 ± 5.1	Mild case: 7.5 ± 1.2 Severe case: 7.3 ± 0.90	100%
Gao et al. (19)	January 23, 2020 to February 2, 2020	Retrospective cohort study	China	43	Mild case: 43.0 ± 14.0 Severe case: 45.2 ± 7.7	26 (60%)	Mild case: 6.0 ± 1.2 Severe case: 7.7 ± 3.4	NR	Mild case: 1 (4%) Severe case: 6 (40%)

*BG, blood glucose; HbA1c, glycated hemoglobin A1c; NR, not reported*.

### Study Quality and Publication Bias

Supplementary Table 2 shows the assessment of quality using Newcastle-Ottawa Scale for three studies included in this meta-analysis. Higher scores indicate better quality. Based on the Newcastle-Ottawa Scale, a maximum of eight points can be awarded to each article. Since only three studies were included in this meta-analysis, a linear regression test of funnel plot asymmetry (Egger's test) could not be carried out.

### Association Between Severity of COVID-19 Infection and Blood Glucose

**Figure 2:**
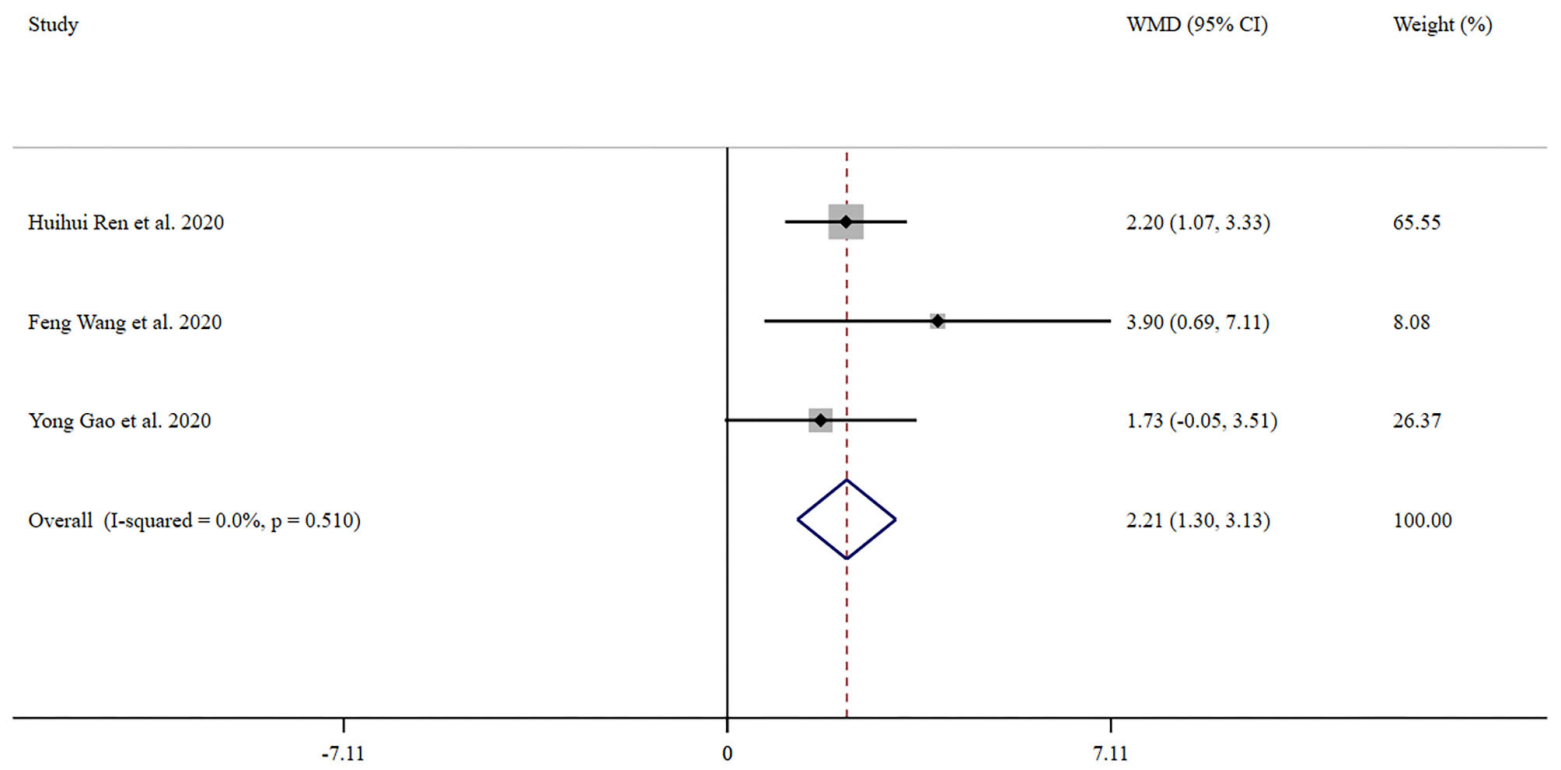
Forest plot of meta-analysis of the effect of severity of COVID-19 on blood glucose. Data are pooled WMDs with 95% CIs. WMD, weighted mean difference; CIs, confidence intervals.

Three trials with 222 patients compared blood glucose between severe group and mild group. Forest plot for the overall effect of severity on blood glucose is presented in Figure 2. The pooled WMD was 2.21 (95% CI: 1.30–3.13). Since the evidence collected in our meta-analysis showed no heterogeneity (*I*^2^ = 0%), a fix-effects model was performed. The z-test result for overall effects was statistically significant (*P* < 0.001), indicating a significantly greater elevation in blood glucose in patients with severe COVID-19 infection than those in the mild group.

### Association of Severity With HbA1c

**Figure 3:**
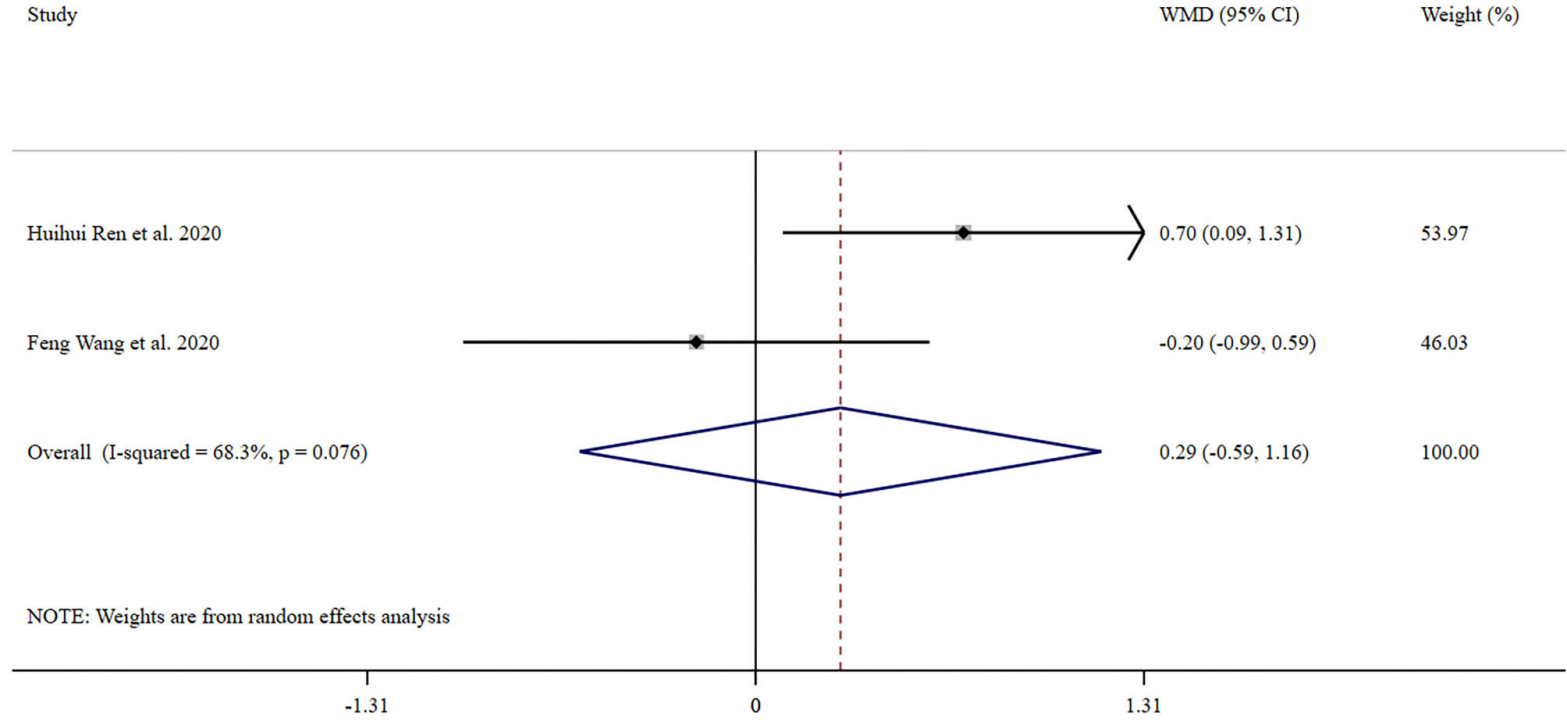
Forest plot of meta-analysis of the effect of severity of COVID-19 on HbA1c. Data are pooled WMDs with 95% CIs. HbA1c, glycated hemoglobin A1c; WMD, weighted mean difference; CIs, confidence intervals.

Two trials reported data on HbA1c both in severe group and mild group. Forest plot for the overall effect of severity on HbA1c is presented in Figure 3. In the overall pooled estimate of two studies with 179 COVID-19 infected patients, the pooled WMD was 0.29 (95% CI: −0.59 to 1.16). Since the evidence collected in our meta-analysis showed heterogeneity (*I*^2^ = 68.3%), a random-effects model was conducted. The z-test result for overall effects showed no statistical significance (*P* = 0.52), which suggested that severity of COVID-19 did not significantly impact HbA1c.

## Discussion

Until now, there are accumulating evidences that diabetes is closely correlated with increased risk of COVID-19, as well as poor outcomes (9, 20). However, it remains unclear regarding the effect of severity of COVID-19 on glycemic parameters. Our analyses found that severe COVID-19 infection was significantly associated with increased blood glucose. Meanwhile, we investigated the correlation between severity and HbA1c. However, we did not provide adequate evidence that patients with severe COVID-19 were more likely to have higher HbA1c than those with mild COVID-19. Since viral infection and hyperglycemia adversely affect each other, this study highlights the need to effectively monitor blood glucose to improve prognosis in patients infected with COVID-19.

Due to defects in innate immunity affecting phagocytosis, neutrophil chemotaxis, and cell-mediated immunity, individuals with diabetes are more likely to have infection (21). A population-based study pointed out that nearly half of patients with diabetes had at least one hospitalization or physician claim because of an infectious disease in each cohort year (22). Additionally, study has demonstrated that people with diabetes are at increased risk for lower respiratory tract infection, urinary tract infection, and skin and mucous membrane infection (23). Furthermore, Kornum et al. suggested that subjects with type 1 diabetes had a 4.4-fold increased risk of a pneumonia-related hospitalization, and subjects with type 2 diabetes displayed a 1.2-fold increased risk of a pneumonia-related hospitalization compared with those without diabetes (24). Recently, COVID-19, caused by SARS-CoV-2, has become a global catastrophe. Numerous studies have been performed to focus on the association between diabetes and COVID-19. Based on the available data, patients with diabetes are more susceptible to COVID-19 than those without diabetes.

However, it is worth noting that infection leads to profound alterations in whole-body metabolism (14). Sustained inflammation affects systemic glucose homeostasis and contributes to hyperglycemia (25). Besides, Šestan et al. reported that the activated immune system drove systemic insulin resistance in response to viral infection (26). Another study found that long-term innate immune activation could impair insulin secretion and action, and play an important role in the pathology of diabetes (27). Therefore, we speculated that there may be a strong relationship between the severity of COVID-19 and glycemic parameters, even in those without diabetes. In the present meta-analysis, we found that blood glucose was significantly higher in patients with severe COVID-19 than those with mild COVID-19 (WMD 2.21, 95% CI: 1.30–3.13, *P* < 0.001, *I*^2^ = 0%). A recent study by Zhang et al. reported that COVID-19 infection induced an increase in blood glucose, even those not diagnosed with diabetes before admission (4). However, this study did not compare blood glucose between severe and mild COVID-19 patients. Guan et al. indicated that the prevalence of diabetes was significantly higher in patients with severe COVID-19 (28). Notably, other studies have demonstrated that hyperglycemia is associated with poor prognoses, while better glycemic control is closely associated with improvement in clinical outcomes in COVID-19 patients (4, 11). Therefore, clinicians should pay more attention to the blood glucose status in patients with COVID-19.

Additionally, our study found that HbA1c was slightly higher in individuals with severe COVID-19 than those with mild COVID-19, yet this difference did not reach significance (*P* = 0.52). However, it is noteworthy that there were only two studies with small sample size that explored the influence of severity of COVID-19 on HbA1c, which might affect the outcomes of interest. Moreover, HbA1c reflects the average blood glucose concentration over the past 2–3 months. Therefore, the effect of short-term viral infection on HbA1c levels may not be prominent. Nevertheless, additional researches with large sample size are needed to verify our results.

The COVID-19 outbreak highlights the importance of understanding the shared diseases pathophysiology with diabetes (29). Over a decade ago, SARS-CoV was detected in pancreas, in addition to lung, suggesting pancreas is the target of coronavirus attack (12). Furtherly, another research found SARS-CoV damaged the endocrine part of pancreas, indicating that SARS-CoV may cause acute insulin dependent diabetes mellitus (13). Noteworthy, pathological changes in pancreas, mainly focal enlargement of the pancreas or dilatation of the pancreatic duct, were observed in patients with severe COVID-19, indicating SARS-CoV-2 may cause pancreatic injury (30). This may be one of the reasons that increased blood glucose was observed in COVID-19 patients without a prior history of diabetes (4).

Furtherly, it was hypothesized that angiotensin converting enzyme 2 (ACE2) may be the key regulator that involved in the association between COVID-19 and hyperglycemia. The main role of ACE2 is to incise angiotensin II to generate angiotensin 1–7 and thereby mediates the protective effects of vasodilation, anti-inflammatory and anti-proliferation. In addition, ACE2 is identified as a receptor that facilitates coronavirus entry into cells (31). A recent research noted that ACE2 expression was substantially increased in patients with diabetes mellitus than those without diabetes (32). Consequently, enhanced susceptibility to COVID-19 infection in patients with diabetes may be attributed to overexpression of ACE2. However, ACE2 expression is not limited to the lung. It has been found in pancreas islets (33), which highlights the need for vigilance in consideration of whether SARS-CoV-2 infection may contribute to the exacerbation or development of diabetes. Individuals with diabetes are more susceptible to COVID-19 than those without diabetes. Meanwhile, SARS-CoV-2 infection could induce hyperglycemia. The pathogenic mechanism of the two may overlap partially, which may be related to ACE2. However, at present, no data are available on ACE2 expression in islets according to the severity of COVID-19. In addition, the study by Ren et al. revealed that triglyceride and glucose index, a marker of insulin resistance, was closely associated with the severity of COVID-19 (17). Insulin resistance may be another explanation for hyperglycemia in patients with COVID-19 infection.

Researches of COVID-19 outbreaks offer important insights that the management of blood glucose is an urgent need. Our study confirmed a strong relationship between the severity of COVID-19 and blood glucose. Future research is required into the effectiveness of improving blood glucose on the prognosis of COVID-19. In addition, this meta-analysis has some limitations. First, we aimed to investigate the association of severity with glycemic parameters in the overall population, including other countries and races. Yet all patients in our meta-analysis were Chinese. Thus, our findings may not be applicable to other regions of the world. Second, although numerous studies have been conducted to focus on the association between diabetes and COVID-19 and we searched all the relevant articles and tried to include as many researches as possible. Studies that provided detailed glycemic parameters according to the severity of COVID-19 were quite limited. It is necessary to update the meta-analysis in the future when more researches were performed to focus on this topic. Third, interpretation of our findings might be limited by the sample size. Therefore, additional researches with large sample size are necessary to confirm our findings. In addition, the presence of diabetes may have an important impact on this association. Besides, it is unclear whether there are differences in the effect of severity of COVID-19 on glycemic parameters between subjects with diabetes and those without diabetes. However, studies included in this meta-analysis were carried out in a mixed population with and without diabetes and no separated data were provided based on diabetes status. Future studies are required to confirm the association of COVID-19 with glycemic parameters in patients with and without diabetes. Finally, details on medications used during COVID-19 infection is not clear. Yet it may have an impact on the results. Future studies should take this into consideration.

## Conclusions

In conclusion, this meta-analysis suggests that sever COVID-19 is associated with increased blood glucose. Attention should be paid to monitor blood glucose status in patients with COVID-19 and better glycemic control may be an important supportive treatment.

## Data Availability Statement

All datasets generated for this study are included in the article/Supplementary Material.

## Author Contributions

JY had full access to all of the data in the study and take responsibility for the integrity of the data and the accuracy of the data analysis. JC and CW contributed to the study concept and design, drafted the manuscript, and performed the statistical analyses. JC, CW, and XW contributed to the acquisition and analysis and interpretation of the data. JC, CW, XW, ZS, and JY critically revised the manuscript. All authors contributed to the article and approved the submitted version.

## Conflict of Interest

The authors declare that the research was conducted in the absence of any commercial or financial relationships that could be construed as a potential conflict of interest.
