# Supplementary material for: The Impact of COVID-19 on Blood Glucose: A Systematic Review and Meta-Analysis
Source: Front Endocrinol (Lausanne). 2020 Oct 5;11:574541. doi: 10.3389/fendo.2020.574541 (PMC7570435; doi:10.3389/fendo.2020.574541)
Supplement: Supplementary file 1 [file Table_1.DOCX]

Supplementary Material

**TABLE 1 |** Search strategies

- **Search strategy in PubMed**

#1 coronavirus [Text Word]

#2 nCoV [Text Word]

#3 HCoV [Text Word]

#4 SARS-CoV-2[Text Word]

#5 COVID [Text Word]

#6 #1 or #2 or #3 or #4 or #5

#7 glucose [Text Word]

#8 glycated Hemoglobin A1c [Text Word]

#9 glycosylated Hemoglobin A1c [Text Word]

#10 HbA1c [Text Word]

#11 #7 or #8 or #9 or #10

#12 #6 and #11

#13 #12 AND ("2019/12/01"[Date - Publication] : "2020/05/15"[Date - Publication])

- **Search strategy in EMBASE**

#1 glucose OR 'glycated hemoglobin a1c' OR 'glycosylated hemoglobin a1c' OR hba1c

#2 ('coronavirus'/exp OR nCoV OR HCoV OR ' SARS-CoV-2' OR COVID) AND [1-12-2019]/sd NOT [16-5-2020]/sd

#3 #1 AND #2

- **Search strategy in Cochrane Library**

#1 coronavirus [All Text]

#2 nCoV [All Text]

#3 HCoV [All Text]

#4 SARS-CoV-2 [All Text]

#5 COVID [All Text]

#6 #1 or #2 or #3 or #4 or #5

#7 glucose [All Text]

#8 glycated Hemoglobin A1c[All Text]

#9 glycosylated Hemoglobin A1c [All Text]

#10 HbA1c [All Text]

#11 #7 or #8 or #9 or #10

**TABLE 2 |** Assessment of the quality of all studies included in the Meta-analysis

|  | **Selection** | **Comparability** | **Measurement** | **Total** |
| --- | --- | --- | --- | --- |
| **Cohort study** | | | | |
| Huihui Ren et al | 4 | 2 | 2 | 8 |
| Feng Wang et al. | 4 | 2 | 2 | 8 |
| Yong Gao et al. | 4 | 2 | 2 | 8 |

**
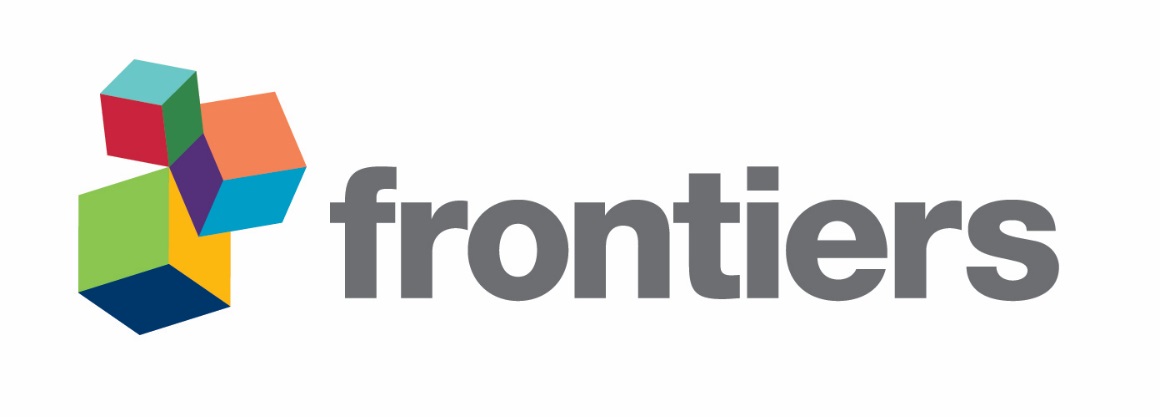
**
